# Solo surgeon ambulatory magnetic-assisted robotic surgery (MARS): initial 51 cases with high patient satisfaction

**DOI:** 10.1007/s00464-025-11879-y

**Published:** 2025-06-11

**Authors:** Graham J. Spurzem, Julio Jimenez, Natasha Paravic, Ignacio Robles, Marcelo Yañez, Gabriel Escalona, Carolina Carmona, Isidora Mendez, Matias Hidalgo, Ryan C. Broderick, Santiago Horgan

**Affiliations:** 1 Department of Surgery, Division of Minimally Invasive Surgery, University of California San Diego, 9300 Campus Point Dr., La Jolla, San Diego, CA 92037 USA; 2 Department of Surgery and Anesthesiology, Hospital Luis Tisné, Santiago, Chile; 3 University of Chile School of Medicine, Santiago, Chile

**Keywords:** Magnetic surgery, Robotic surgery, Ambulatory surgery, Cholecystectomy, New technology

## Abstract

**Background:**

Magnetic-assisted robotic surgery (MARS) is a new platform developed to maximize the benefits of minimally invasive surgery for patients while enhancing surgeon control and visualization. The system is composed of two robotic arms that enable surgeon control of the laparoscopic camera and a deployable intraperitoneal magnetic grasper designed to provide incisionless retraction. The aim of this study was to evaluate the outcomes of the MARS platform following its use in outpatient laparoscopic cholecystectomy by a solo surgeon and examine patient perceptions of this approach.

**Methods:**

A retrospective review of a prospectively maintained database identified all patients who underwent outpatient reduced port laparoscopic cholecystectomy assisted by MARS for symptomatic cholelithiasis from January 2024 to August 2024 at a tertiary care hospital. All cholecystectomies were performed without a surgical assistant. Primary outcomes were 30-day morbidity, 30-day readmission, operative time, and 30-day patient satisfaction as measured by a modified version of the Surgical Satisfaction Questionnaire (SSQ).

**Results:**

Fifty-one patients were identified. Mean age was 47.3 ± 13.9 years and most patients were female (N = 45, 88.2%). Mean operative time was 52.8 ± 16.3 min. All patients were discharged within 6 h after surgery. There were no 30-day morbidities or readmissions. Forty patients (78.4%) completed the modified SSQ at 30 days postoperatively and reported high degrees of satisfaction with pain management, return to preoperative baseline function, and the overall surgical experience. 92.5% of patients were “very satisfied” with the use of the MARS system and 97.5% would recommend use of the system to others.

**Conclusion:**

This study demonstrates the safety and feasibility of the MARS platform for use in outpatient reduced port laparoscopic cholecystectomy by a solo surgeon with high patient satisfaction. MARS was particularly useful for simultaneous gallbladder retraction and laparoscopic camera manipulation by the operating surgeon, thus eliminating the need for a surgical assistant.

Over the last four decades, advances in perioperative care and the widespread adoption of minimally invasive surgery (MIS) have significantly reduced the amount of time patients spend in the hospital recovering from surgical procedures. Outpatient surgery has become an increasingly important component of surgical care in the United States (US) and worldwide as a result. Ambulatory surgery centers (ASC) have grown rapidly in response to these trends and have been shown to provide high-quality cost-effective care [1, 2]. With outpatient surgical volume expected to grow 18% by 2033, there is a need for innovative solutions to address increasing demand while preserving system efficiency [3]. In addition, general surgeons are expected to be significantly impacted as several minimally invasive procedures continue to shift to the outpatient setting [4–6].

A number of minimally invasive techniques have been introduced since the advent of laparoscopy with the goal of reducing postoperative pain and improving cosmesis, including magnetic-assisted surgery [7]. The concept of magnetic surgery involves deploying a magnetic instrument into the peritoneal cavity that is then manipulated by the surgeon using a magnet placed externally onto the abdominal wall, thus eliminating a laparoscopic port. The first magnetic system to receive Food and Drug Administration (FDA) approval was the Levita® Magnetic Surgical System (Levita Magnetics, Mountain View, CA), which has proven safety and efficacy in laparoscopic cholecystectomy, bariatrics, urology, and colorectal surgery [8–10]. The magnetic-assisted robotic surgery (MARS®) platform was subsequently developed by Levita, composed of two robotic arms that enable control of an external magnet and laparoscopic camera by the operating surgeon. In a recent series of minimally invasive urologic procedures, the MARS platform was particularly useful for tissue retraction, avoiding additional incisions, and reducing the need for a surgical assistant [11].

The practice of robotic-assisted reduced port laparoscopic surgery by a solo surgeon may be uniquely poised to meet the demands of increasing outpatient surgical volume. Routine use of MARS reduces the number of required laparoscopic ports, thus leading to less tissue trauma and postoperative pain, which may help facilitate same-day discharge [12]. Reducing the need for additional surgical assistants may also provide a net cost benefit and improve operative efficiency by enhancing surgeon control. The aim of this study was to evaluate the outcomes of the MARS platform following its use during outpatient reduced port laparoscopic cholecystectomy by a solo surgeon and examine patient perceptions of this novel approach.

## Methods

A retrospective review of a prospectively maintained database identified all patients who underwent outpatient reduced port laparoscopic cholecystectomy assisted by MARS for treatment of symptomatic cholelithiasis between January 2024 and August 2024 at a tertiary care hospital located in Santiago, Chile. All cholecystectomies were performed independently by four experienced laparoscopic surgeons without a surgical assistant. Each surgeon had participated in the clinical trial that led to eventual FDA clearance of the robotic system and were experienced in using the robot. The learning curve for each surgeon on the system was comparable to the results published by Larenas et al., who found that the learning curve of the system for use in urologic procedures was four cases [11]. Ethics approval was obtained from the Scientific Ethics Committee of the Metropolitan Eastern Health Service prior to the start of the study (approval code: SSMOriente291024-6).

### MARS platform

**Fig. 1:**
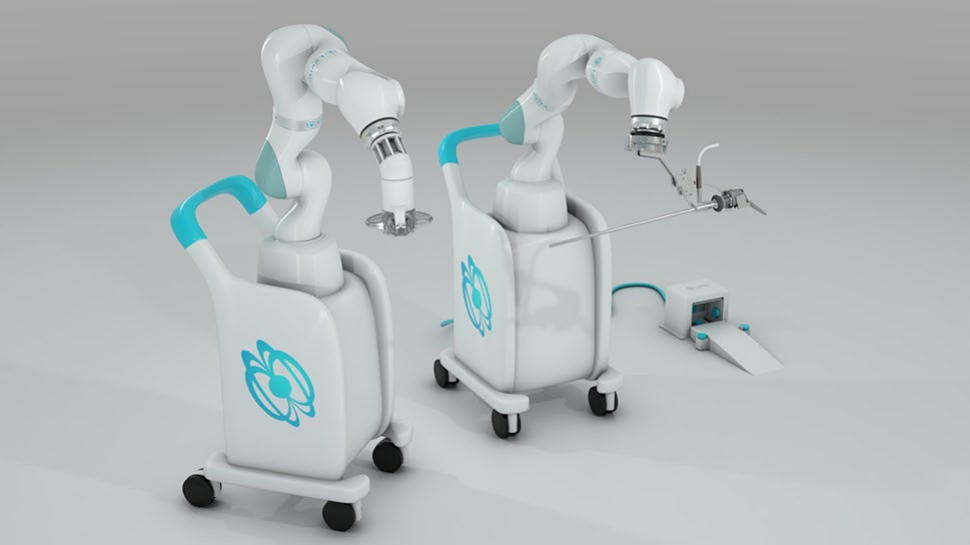
The magnetic-assisted robotic surgery (MARS) platform is composed of two independent robotic arms: the magnetic arm holds the external controller of the magnetic system (left) and the camera arm holds the laparoscope (middle). Both arms are controlled by a foot pedal (right) that contains forward/backward, right/left, up/down buttons, and a side button to switch between robotic arms. Each robotic arm is mounted on a wheeled cart

**Fig. 2:**
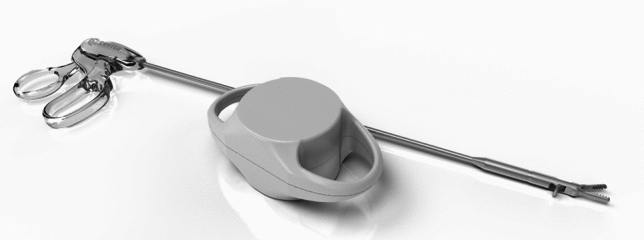
Magnetic grasper device comprised a laparoscopic handle and detachable magnetic grasper at the distal end. The external magnetic controller is also shown

**Fig. 3:**
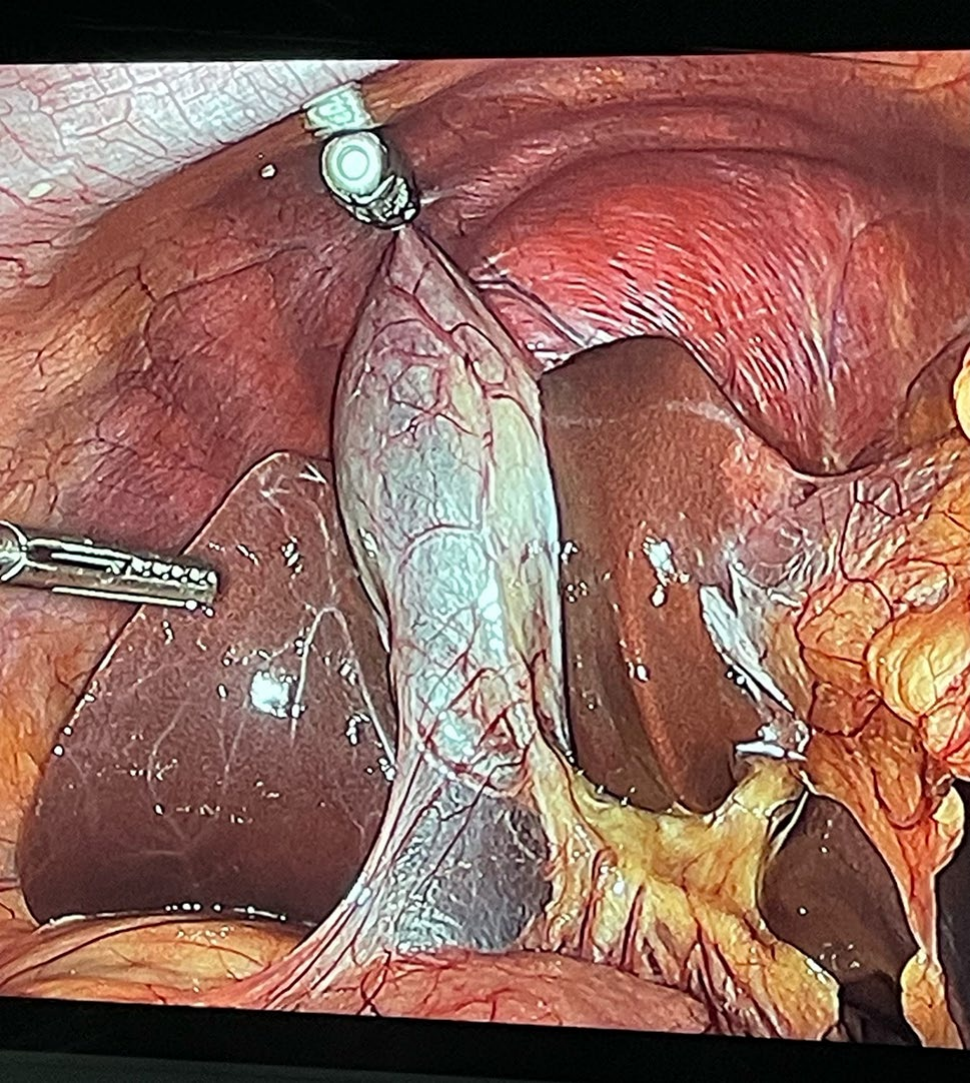
Operative image of the detachable magnetic grasper attached to the gallbladder (top of image), enabling incisionless retraction with the external robotic arm magnet

**Fig. 4:**
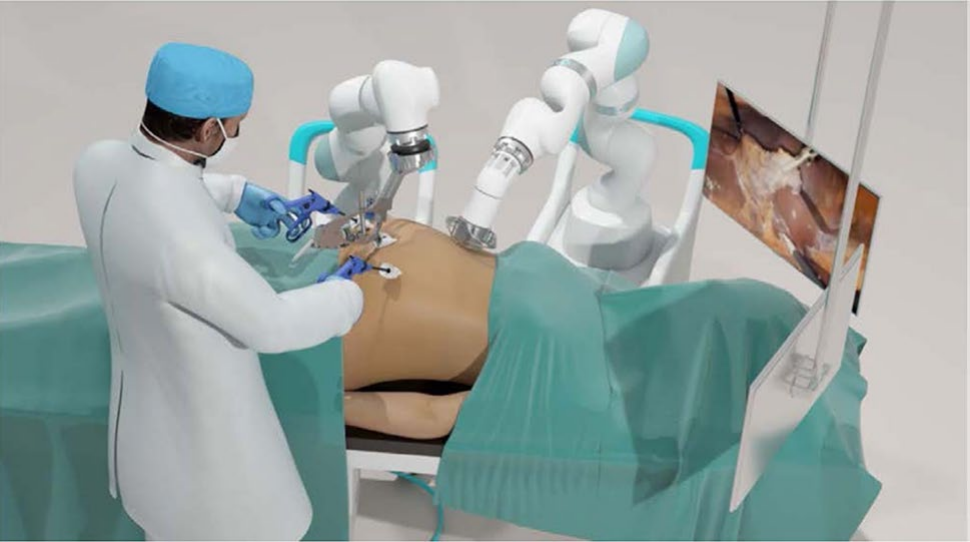
Illustration of surgeon performing independent surgery with the magnetic-assisted robotic surgery (MARS) platform

**Fig. 5:**
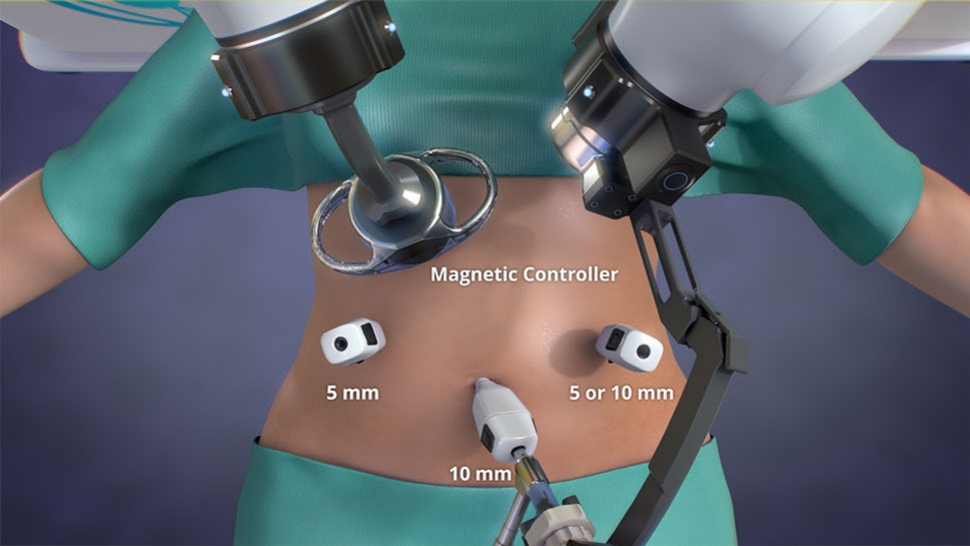
Reduced port technique used for each cholecystectomy (utilizing 3 ports instead of 4) with the magnetic controller replacing the standard epigastric port for gallbladder retraction

The MARS platform consists of two independent robotic arms controlled by a surgeon foot pedal, each with seven degrees of freedom (Fig. 1). The magnetic arm controls an external magnet, while the camera arm holds an off-the-shelf laparoscope. Each robotic arm features a graphical user interface for system setup and is mounted on a wheeled cart. The system also includes a laparoscopic single-use magnetic hand piece with a deployable atraumatic magnetic grasper attached to its distal end (Fig. 2). The magnetic grasper is introduced into the peritoneal cavity through a 10 mm trocar and attached to the desired tissue. Once the tissue is firmly grasped, the laparoscopic handle is squeezed to release the magnetic grasper (Fig. 3). The external magnet on the robotic arm is then joined with the intraperitoneal magnetic grasper. The surgeon can then manipulate the position of the grasper using the foot pedal that controls the magnetic robotic arm. Tissue handling is maintained by magnetic attraction between the external magnet on the robotic arm and the intraperitoneal magnetic grasper. The MARS platform is designed to enable simultaneous surgeon control of the laparoscope and magnetic grasper, thus reducing the need for a surgical assistant (Fig. 4). A reduced port technique was used for each case (utilizing 3 ports instead of 4) with the magnetic grasper replacing the standard epigastric port for gallbladder retraction (Fig. 5).

### Outcomes

Patient demographic data including age and gender were collected. The primary outcomes of interest were 30-day complications, 30-day readmission, operative time, and patient satisfaction with the MARS system. For postoperative pain control, each patient received ketoprofen 100 mg IV in the post-anesthesia care unit and were discharged with paracetamol 1 g PO every 8 h for 5 days and ketoprofen 50 mg PO every 8 h for 3 days.

Patient satisfaction was measured using a modified version of Surgical Satisfaction Questionnaire (SSQ-8). SSQ-8 is a validated eight-item survey designed to measure patient satisfaction after surgery [13]. For the first 9 questions, responses were recorded on a 5-point Likert scale (1 = “very unsatisfied,” 5 = “very satisfied”) and a 3-point Likert scale was used for the last 2 questions (1 = “no,” 2 = “neutral,” 3 = “yes”). Questions 1 and 2 related to postoperative pain control, while questions 3, 4, and 5 related to return of preoperative baseline function. Questions 6, 10, and 11 related to overall satisfaction with the surgical experience. In this study, the standard SSQ-8 was modified to include 3 additional questions (numbered 7, 8, and 9) specific to MARS, recorded in a similar 5-point Likert scale. The questionnaire was administered 30 days postoperatively during the routine follow-up appointments and recorded in each patient’s chart.

### Statistical analysis

Responses from the patient satisfaction survey were grouped into 3 categories for questions 1 through 9: satisfied (including “very satisfied” and “satisfied” responses), neutral, and unsatisfied (including “very unsatisfied” and “unsatisfied” responses). Questions 10 and 11 maintained their original categorization (“yes,” “neutral,” and “no”). Continuous variables were reported as mean ± standard deviation (SD). Categorical variables were reported as a frequency and percentage. Statistical analyses were performed using Python (version 3.11.5).

## Results

A total of 51 outpatient laparoscopic cholecystectomies were performed using the MARS platform. Mean patient age was 47.3 ± 13.9 years and the majority of patients were female (*N* = 45, 88.2%). Mean operative time was 52.8 ± 16.3 min. All patients were discharged within 6 h after surgery. There were no major 30-day postoperative complications or readmissions. During the first 6 h postoperatively, 5 patients (9.8%) experienced nausea and vomiting that was managed with antiemetics. One patient (2.0%) had minor incisional bleeding that was controlled with manual compression. During the 30-day follow-up period, 3 patients (5.9%) experienced self-limited diarrhea and 2 patients (3.9%) developed minor incisional bruising.

Table 1 details the results of the modified patient satisfaction survey. Forty patients (78.4%) completed the survey at 30 days postoperatively. Regarding postoperative pain management, 87.5% of participants were satisfied, with only 5% expressing dissatisfaction with pain control in the hospital and at home. Similarly, most patients were satisfied with the amount of time needed to return to normal daily activities (90.0%), work (90.0%), and a normal exercise routine (95.0%). 95.0% of patients were satisfied with the results of surgery, 87.5% would undergo the surgery again, and 97.5% would recommend the surgery to someone else.

**Table 1:** Results of the modified Surgical Satisfaction Questionnaire (N = 40 respondents). Responses in each category are reported as a frequency and percentage.

Number	Question	Very satisfied	Satisfied	Neutral	Unsatisfied	Very unsatisfied
1	How satisfied are you with how your pain was controlled in the hospital after surgery?	31 (77.5%)	4 (10.0%)	3 (7.5%)	2 (5.0%)	0
2	How satisfied are you with how your pain was controlled when you returned home after surgery?	25 (62.5%)	10 (25.0%)	3 (7.5%)	1 (2.5%)	1 (2.5%)
3	How satisfied are you with the amount of time it took for you to return to your daily activities, for example, housework or social activities outside of the home?	31 (77.5%)	5 (12.5%)	2 (5.0%)	1 (2.5%)	1 (2.5%)
4	How satisfied are you with the amount of time it took for you to return to work?	32 (80.0%)	4 (10.0%)	1 (2.5%)	2 (5.0%)	1 (2.5%)
5	How satisfied are you with the amount of time it took for you to return to your normal exercise routine?	34 (85.0%)	4 (10.0%)	0	1 (2.5%)	1 (2.5%)
6	How satisfied are you with the results of your surgery?	37 (92.5%)	1 (2.5%)	2 (5.0%)	0	0
7	What do you think about your surgeon using a new robot that reduces incisions for your surgery?	37 (92.5%)	2 (5.0%)	1 (2.5%)	0	0
8	Are you satisfied with the number of scars/ports on your body after surgery?	37 (92.5%)	2 (5.0%)	0	1 (2.5%)	0
9	How satisfied are you with the care provided by your surgeon?	33 (82.5%)	2 (5.0%)	3 (7.5%)	0	2 (5.0%)

		Yes	Neutral	No
10	Looking back, if you “had to do it all over again” would you have the surgery again?	35 (87.5)	2 (5.0)	3 (7.5)
11	Would you recommend this surgery to someone else?	39 (97.5)	1 (2.5)	0

The additional MARS-specific questions also showed strong positive feedback. 97.5% of patients were satisfied with their surgeon using a new robotic system designed to reduce the number of incisions. 87.5% of patients were satisfied with the care provided by their surgeon, with a small percentage expressing neutral or unsatisfied responses. Of the patients who were neutral or unsatisfied with their surgical experience, five were able to be contacted for further clarification. Four patients had administrative issues related to discharge and problems with follow-up scheduling. One patient would not recommend the procedure due to experiencing postoperative diarrhea during the three weeks following surgery.

## Discussion

This study demonstrates the safety and feasibility of the MARS platform for use in outpatient reduced port laparoscopic cholecystectomy by a solo surgeon with high patient satisfaction. Of the 51 procedures performed with MARS, there were no postoperative complications or readmissions within 30 days. In addition, operative efficiency was maintained with MARS despite the lack of a surgical assistant, with a mean operative time of less than 60 min. This was likely enabled by the ability of the operating surgeon to simultaneously control the laparoscopic camera and gallbladder retraction with the robot as needed. Our study builds upon previous literature demonstrating the utility of magnetic-assisted surgery in reduced port MIS and is the first to report a cholecystectomy cases series performed by a solo surgeon with the MARS system.

Surgical procedures in the US and abroad are increasingly shifting to outpatient and non-hospital locations. According to the 2024 US Ambulatory Surgery Center Market Report published by the Health Industry Distributors Association (HIDA), the 2024 ASC market is valued at $45.6 billion and projected to grow 21% to $55.3 billion by 2029 as procedures become less invasive. Outpatient surgical volumes are projected to reach 109.6 million cases by 2033, representing an 18% increase from 2023 levels [14]. Technological advances, increasing patient demand, and potential cost savings are driving the shift of numerous procedures across specialties to the outpatient setting. This shift also comes amid critical staffing shortages, particularly among nurses and surgical technologists, which has prompted facilities to adopt strategies like workflow automation and flexible scheduling to attract and retain staff. The 2024 HIDA report stated that nearly 70% of ASC leaders reported increased difficulty recruiting staff in 2023 compared to 2022. Hospitals have also faced a decrease of approximately 94,000 healthcare workers since February 2020, with vacancy rates for nurses increasing by up to 30% in some locations [15]. Robotic surgery may offer a partial solution to these challenges by reducing the need for specialized surgical staff, alleviating some pressure caused by workforce shortages while addressing growing procedural demands. Enabling surgeons to perform solo procedures with a surgeon-controlled robotic assistant will likely aid in these efforts.

Several studies have demonstrated the use of robotic surgical systems to perform solo surgery, particularly robotic camera holders [16–22]. These investigations have shown that robotic camera assistance is safe and feasible for a range of minimally invasive procedures, including laparoscopic cholecystectomy, hysterectomy, colon resection, bariatrics, and foregut surgery. Robotic camera holders can provide a stable view of the operative field and may also improve surgeon ergonomics [23, 24]. It follows that eliminating the need for a camera assistant could reduce the amount of time spent in a suboptimal field of view, unintended rotation of the camera horizon, tissue contact, tremor, and undesired camera movements. Two randomized controlled trials evaluated the application of a robotic camera holder on cholecystectomy specifically and found that robotic holders were practical, reliable, and can reduce operative personnel [16, 25]. Previous studies have also shown that solo surgery with robotic camera holders can be cost beneficial [16–20, 26, 27]. Stott et al. found that the use of a robotic camera holder provided cost savings compared with surgical trainees and nurse assistants during laparoscopic liver resections [28]. In addition to providing camera control, the MARS system enables concurrent surgeon control of tissue retraction, permitting even more precise intraoperative maneuvers and visualization. Further studies are warranted to investigate the influence of MARS on operative efficiency compared to standard laparoscopy, particularly regarding operative time and economy of motion.

Effective pain management is an essential component of postoperative care. Inadequately controlled pain negatively affects quality of life and functional recovery and can potentially require unplanned inpatient admission after ambulatory surgery [29, 30]. Persistent incisional pain in particular is a common and problematic postoperative complication occurring in approximately 1 in 30 patients [31]. The majority of patients who develop persistent incisional pain report interference across aspects of daily life and more than half will seek analgesic use, which can result in increased medication side effects and personal healthcare costs. Efforts to reduce the size and number of incisions required to perform surgery have been a key driving force in surgical innovation, from advanced endoscopic techniques to natural orifice transluminal endoscopic surgery (NOTES) [32]. The MARS system’s use of magnetic attraction to provide incisionless retraction represents another advancement. Welsh et al. used the Levita magnetic system in place of an externally mounted Nathanson liver retractor in a series of minimally invasive bariatric procedures and found that use of the magnetic system resulted in decreased postoperative pain scores and decreased hospital length of stay compared to a control cohort [12]. Previous studies of this magnetic system in laparoscopic cholecystectomy have also demonstrated low postoperative pain scores and no apparent trauma to the abdominal wall from the external magnet at 6 h postoperatively [7]. While we do not directly compare postoperative pain control with the MARS system to standard laparoscopy, the majority of patients in this study were satisfied with their pain control in the hospital and at home. Additional studies are necessary to rigorously investigate the pain control benefits of reduced port laparoscopic surgery across a range of procedures and patient populations.

Patient satisfaction is increasingly recognized as an important metric that influences both postoperative outcomes and healthcare reimbursement models. Studies have shown that patients with higher satisfaction levels are more likely to adhere to postoperative care instructions, report reduced levels of stress, and experience fewer complications and readmissions, all of which contribute to improved postoperative recovery [33–35]. Moreover, efficient and high-quality surgical care is associated with higher patient satisfaction scores [33]. Insurance providers and healthcare systems have incorporated patient satisfaction metrics into physician and hospital reimbursement structures, emphasizing the need for patient-centered care. Notably, in 2012, the Department of Health and Human Services tied a percentage of Medicare reimbursement to patient satisfaction scores as measured by the Hospital Consumer Assessment of Healthcare Providers and Systems (HCAHPS) survey, thus incentivizing hospitals to improve patient perceptions of the care they receive [36]. Robotic surgery and other technological advances like the MARS system may be utilized to enhance the surgical experience for patients. In a prospective study of 51 patients who underwent minimally invasive bariatric surgery with the magnetic system, 90% of patients believed that reduced incision magnet-assisted surgery would provide superior cosmetic results compared to standard laparoscopy [37]. The results of our survey further support these findings, as the overwhelming majority of patients in our cohort were satisfied with the use of new technology designed to reduce surgical incisions. The majority of patients in our study were also satisfied with the surgical experience overall.

This study is limited by its retrospective, single-center design, and lack of a direct comparison to standard laparoscopy. In addition, all cholecystectomies were performed by surgeons with extensive experience using the MARS system, which could limit the generalizability of our findings. We also did not objectively account for additional pain medications patients may have requested postoperatively outside of the standard regimen. However, we plan to conduct a randomized prospective study to compare postoperative pain between the reduced port technique with MARS and the conventional four-port technique for laparoscopic cholecystectomy. Additional work is also needed to validate our modifications to the SSQ-8. Further studies are warranted to evaluate the operative efficiency, learning curve, and cost-effectiveness of the MARS system for solo laparoscopic cholecystectomy. Use of the system for solo surgery should also be investigated for additional procedures across surgical disciplines.

## Conclusion

This study is the first to demonstrate the safety and feasibility of the MARS platform for use in outpatient reduced port laparoscopic cholecystectomy by a solo surgeon with high patient satisfaction. MARS was particularly useful for simultaneous gallbladder retraction and laparoscopic camera manipulation by the operating surgeon, thus eliminating the need for a surgical assistant. These results also showcase the potential benefits of the MARS platform for other surgical procedures. Furthermore, due to high patient satisfaction with this technique, MARS may improve the patient experience and strengthen the patient–surgeon relationship.
